# Correlation of R2* with fat fraction and bone mineral density and its role in quantitative assessment of osteoporosis

**DOI:** 10.1007/s00330-023-09599-9

**Published:** 2023-04-05

**Authors:** Zhenghua Liu, Dageng Huang, Yonghong Jiang, Xiaowen Ma, Yuting Zhang, Rong Chang

**Affiliations:** 1 Department of Radiology, Honghui Hospital Affiliated Xi’an Jiaotong University, No. 555, Youyi East Road, Xi’an 710054, China; 2 Department of Spinal Surgery, Honghui Hospital Affiliated Xi’an Jiaotong University, No. 555, Youyi East Road, Xi’an 710054, China

**Keywords:** Magnetic resonance imaging, Bone density, Osteoporosis, Bone marrow, Adipose tissue

## Abstract

**Objectives:**

To investigate the correlation of R2* with vertebral fat fraction (FF) and bone mineral density (BMD), and to explore its role in the quantitative assessment of osteoporosis (OP).

**Methods:**

A total of 83 patients with low back pain (59.77 ± 7.46 years, 30 males) were enrolled, which underwent lumbar MRI in IDEAL-IQ sequences and quantitative computed tomography (QCT) scanning within 48h. The FF, R2*, and BMD of all 415 lumbar vertebrae were respectively measured. According to BMD, all vertebrae were divided into BMD normal, osteopenia, and OP groups, and the difference of FF and R2* among groups was analyzed by one-way ANOVA. The correlation between R2*, FF, and BMD was analyzed by Pearson’s test. Taking BMD as the gold standard, the efficacies for FF and R2* in diagnosis of OP and osteopenia were assessed by receiver operating characteristic curve, and their area under the curve (AUC) was compared with DeLong’s test.

**Results:**

The FF and R2* were statistically different among groups (*F* values of 102.521 and 11.323, both *p* < 0.05), and R2* were significantly correlated with FF and BMD, respectively (*r* values of −0.219 and 0.290, both *p* < 0.05). In diagnosis of OP and osteopenia, the AUCs were 0.776 and 0.778 for FF and 0.638 and 0.560 for R2*, and the AUCs of R2* were lower than those of FF, with *Z* values of 4.030 and 4.087, both *p* < 0.001.

**Conclusion:**

R2* is significantly correlated with FF and BMD and can be used as a complement to FF and BMD for quantitative assessment of OP.

**Key Points:**

• R2* based on IDEAL-IQ sequences has a definite but weak linear relationship with FF and BMD.

• *FF is significantly correlated with BMD and can effectively evaluate BMAT.*

• R2* can be used as a complement to FF and BMD for fine quantification of bone mineral loss and bone marrow fat conversion.

## Introduction

Osteoporosis (OP) is an age-increasing disease that seriously affects the health of the elderly, and its diagnosis and evaluation rely mainly on bone mineral density (BMD) measurements [1, 2]. Recent studies have shown that bone marrow adipose tissue (BMAT) plays an important role in the development and progression of osteoporosis (OP), and may be a biomarker for OP [3–5]. Further studies have pointed out that differences in BMAT amounts may reduce the accuracy of BMD and that BMAT should be quantified to correct for BMD [6, 7].

Magnetic resonance imaging (MRI) has clear advantages in quantifying bone marrow composition [8], such as iterative decomposition of water and fat with echo asymmetry and least-squares estimation quantitation (IDEAL-IQ) sequences [9, 10]; based on multi echo acquisition, it can obtain fat fraction (FF) imaging, R2* imaging, fat imaging, and water imaging through one scan [11]. In Ergen’s study [12], a significant negative correlation was found between FF and BMD of the vertebral body, suggesting that loss of vertebral bone mineral can be assessed using FF. In Ji’s study [13], FF and R2* were used to quantify the vertebral BMAT and found that the BMAT was associated with degeneration of the adjacent discs.

R2*, the inverse of the effective transverse relaxation time [T2*], is a derived research product that has received increasing attention in recent studies. In the spine, R2* has been tried for the differentiation of osteoporotic, traumatic, and malignant vertebral fractures [14–16] and also to distinguish aplastic anemia from myelodysplastic syndromes [17]. Some other studies have pointed out that R2* (T2*) of the vertebral bone marrow correlates with the ferritin content of the red bone marrow as well as the density and orientation of the trabeculae [18, 19].

We were interested in the correlation between R2* and BMAT content of the vertebral body as well as BMD. However, the relationship between R2* with FF and BMD has been rarely reported and remains controversial [18, 20, 21], and its role in the quantitative assessment of OP still needs further validation. In this study, we enrolled a group of patients with chronic low back pain who underwent IDEAL-IQ sequences scan of the lumbar spine as well as QCT scan, and we aimed to investigate the correlation between R2* with FF and BMD, and to explore its role in the quantitative assessment of OP.

## Materials and methods

### Study design

**Fig. 1:**
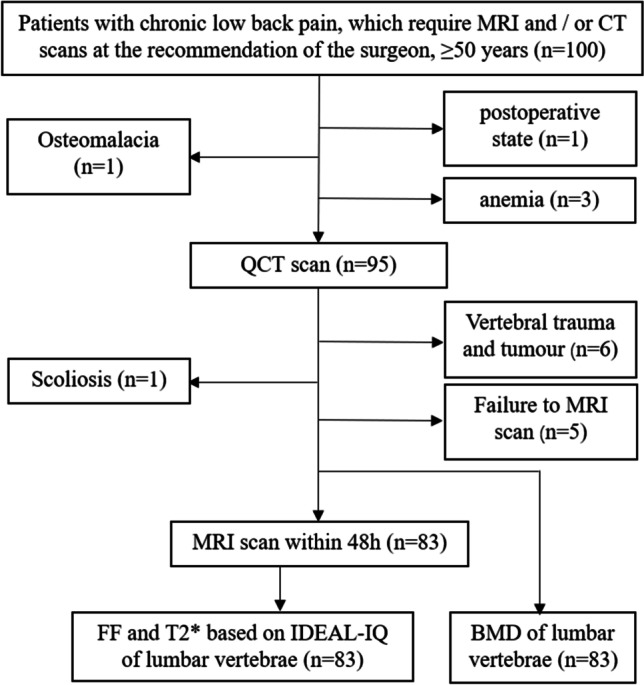
The flowchart of the study. CT, computed tomography; QCT, quantitative computed tomography; MRI, magnetic resonance imaging; FF, fat fraction; BMD, bone mineral density

The present study was conducted following the Declaration of Helsinki (as revised in 2013) and approved by the Ethics Committee of our hospital (IRB No. 201902068). This is a secondary analysis of a prospective study, and some patients with chronic low back pain received MRI and QCT scans at the recommendation of surgeons and were initially included in the study. Inclusion criteria were as follows: (1) age should be ≥50 years; (2) lumbar MRI and QCT scan within 48 h. Exclusion criteria included the following points: (1) scoliosis; (2) localized osteosclerosis in vertebral cancellous bone; (3) vertebral trauma and tumor; (4) metabolic and hematopoietic system diseases other than osteoporosis; (5) postoperative state of lumbar vertebra. The flowchart displaying patient inclusion of this study is shown in Fig. 1.

### MRI scanning

MRI examinations relied on a 3.0-T superconducting MR scanner (Discovery 750, GE Healthcare), with standard human body coil and sagittal scanning. Prior to IDEAL-IQ, T1-weighted image (T1WI) (repetition time (TR)/time to echo (TE) = 400/13 ms), T2WI (TR/TE = 2500/102 ms), FOV 36cm ×36 cm, matrix of 224 × 192, pixel size 1.6 mm × 1.9 mm, slice thickness of 3 mm, intersection gap of 0.4, number of excitations (NEX) of 1; IDEAL-IQ: TR of 7.4 ms, minimum TE of 1.3 ms, maximum TE of 5.3 ms, flip angle of 4°, echo train length of 5, bandwidth of 111.1 kHz, and other settings were the same as above. Four group images were acquired in once scanning with IDEAL-IQ sequence: pure water image, pure fat image, fat fraction image, and R2* relaxation rate image.

### Image analysis

**Fig. 2:**
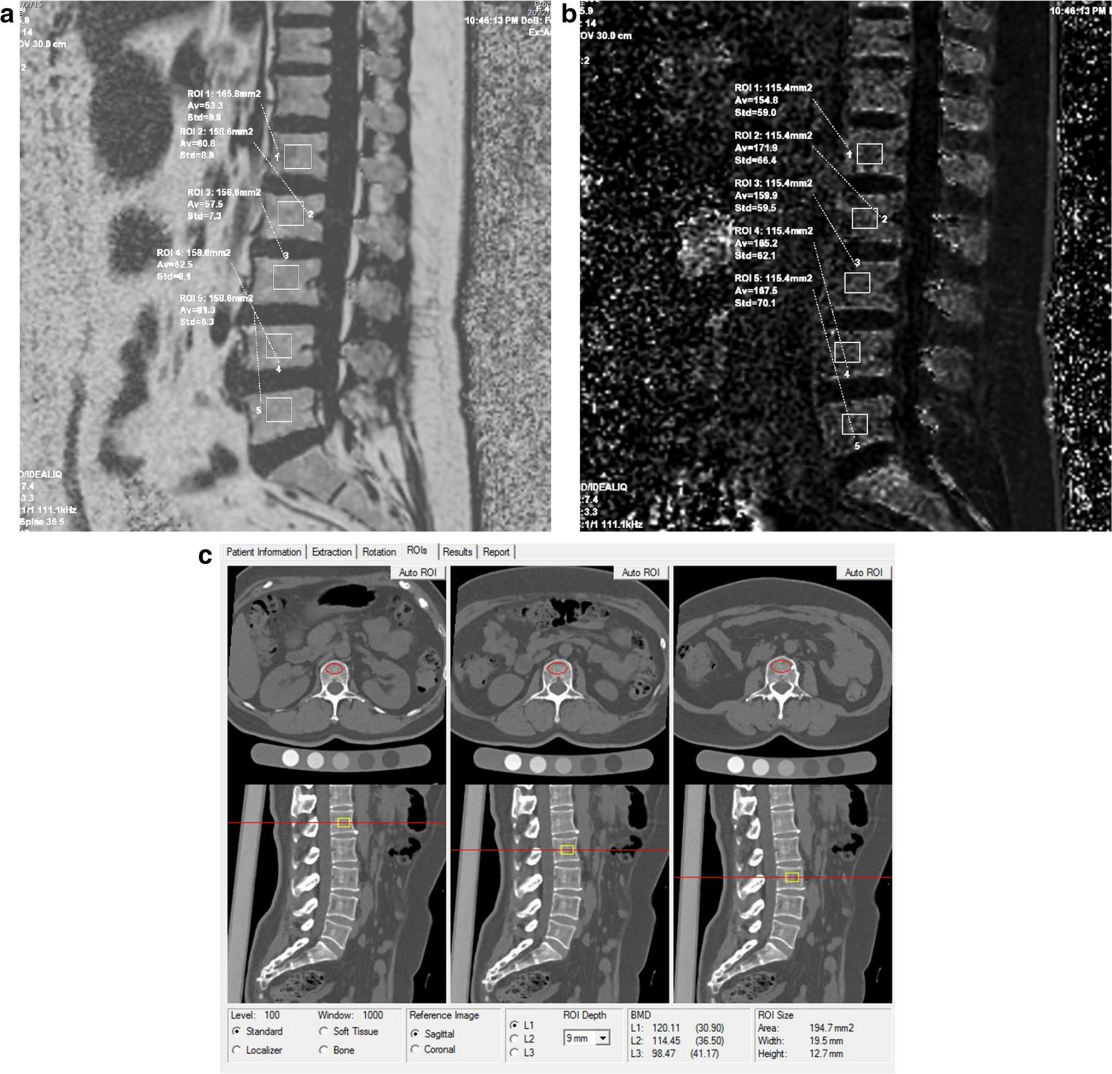
Measurement of FF, R2*, and BMD**. a** FF image of IDEAL-IQ. **b** R2* relaxation rate image of IDEAL-IQ. **c** Measurement of QCT-based BMD

The measurement of FF and R2* was performed on the viewer module of ADW 4.7 workstation. Select the FF image and R2* relaxation rate image respectively, draw a rectangular region of interest (ROI) on the first 2/3 of the vertebral body in the median sagittal diagram, avoiding the vertebral vein sulcus; then, the FF and R2* values of 1st to 5th lumbar vertebrae were measured successively at one slice (Fig. 2a, b). All vertebral measurements were performed independently by two doctors with more than 8 years of experience in musculoskeletal radiology, and take the mean value of 2 measurers as the final value.

### QCT-based BMD measurement and grouping

CT equipment (Somatom Definition Flash; Siemens Healthineers) and QCT analytics (QCT Pro v5.0; Mindways) were calibrated in advance using a quality control phantom. Constant X-ray tube and reconstruction parameter setting were used. A standard QCT corrected phantom was placed under the waist during CT procedure, and the scan data was imported to the QCT analytics. The system generated ROIs in the cancellous bone region of the vertebrae, and the BMD values of the 1st to 5th lumbar vertebrae were measured in sequence (Fig. 2c). According to the BMD values, all vertebrae were divided into BMD normal group (BMD > 120 mg/cm^3^), osteopenia group (120 mg/cm^3^ ≥ BMD > 80 mg/cm^3^), and OP group (BMD ≤  80 mg/cm^3^) [22].

### Statistical analysis

All computations were powered by MedCalc (version 19.0, MedCalc Software) and expressed as the mean ±  standard deviation. Consistency analyses for the measurements of the two readers were performed using the intraclass correlation coefficient (ICC) (ICC of <  0.4 means poor consistency, ICC of 0.4~0.75 means general consistency, ICC of > 0.75 means good consistency). Variables were tested for normality of distribution using the Shapiro-Wilk test. Differences between groups were determined using one-way ANOVA and Tukey-Kramer’s test. Pearson’s test was used to determine the correlation between FF, R2*, and BMD. Taking BMD as the gold standard, the efficacies of FF and R2* for the diagnosis of OP and osteopenia were assessed by receiver operating characteristic (ROC) curve, and their area under the curve (AUC) was compared with DeLong’ test. The significance for all tests was set at *p* value <  0.05.

## Results

### General information

**Fig. 3:**
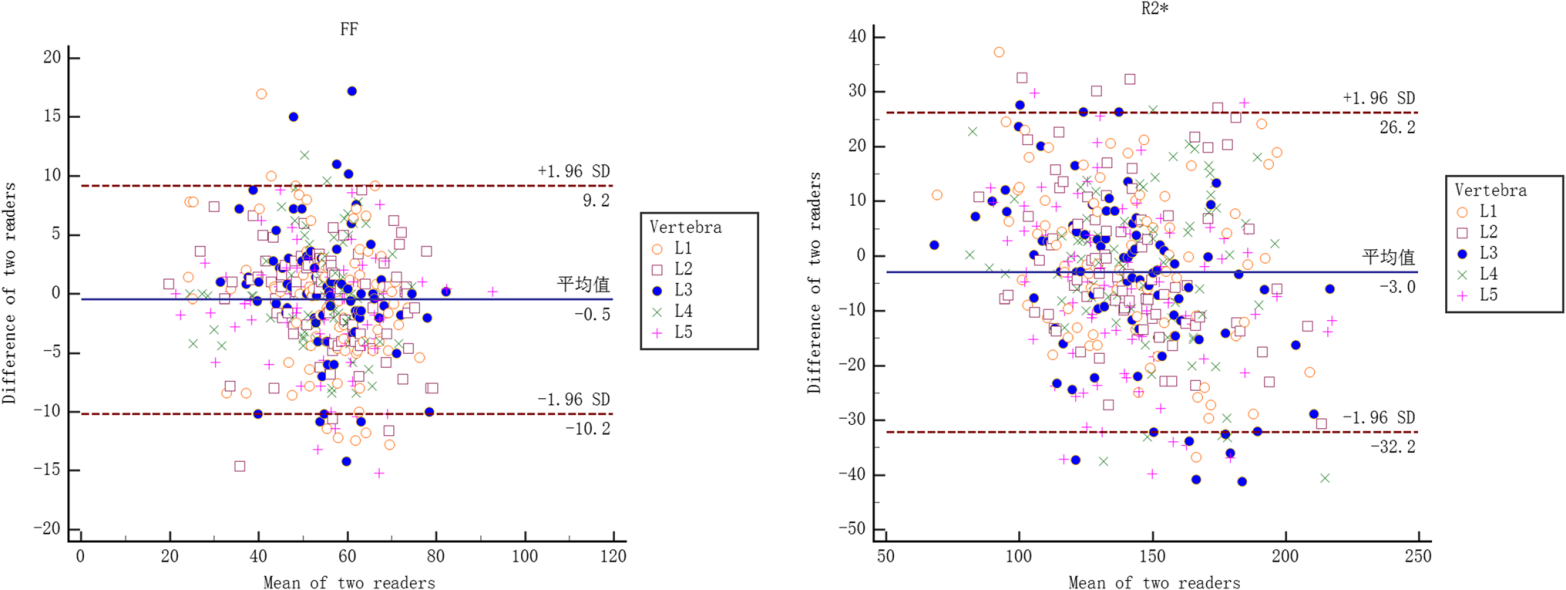
Bland-Altman plots of two readers for FF and R2* values

A total of 83 patients were enrolled in this study. Of them, 30 were males and 53 were females, aged from 50 to 88 years, with an average age of 59.77 ± 7.46 years. The FF and R2* values of all 415 lumbar vertebrae in 83 patients were measured by two doctors, which were in good agreement (ICC = 0.917, 0.886, respectively). The corresponding Bland-Altman plots are shown in Fig. 3, which indicate a reliable agreement between them.

### Grouping

**Fig. 4:**
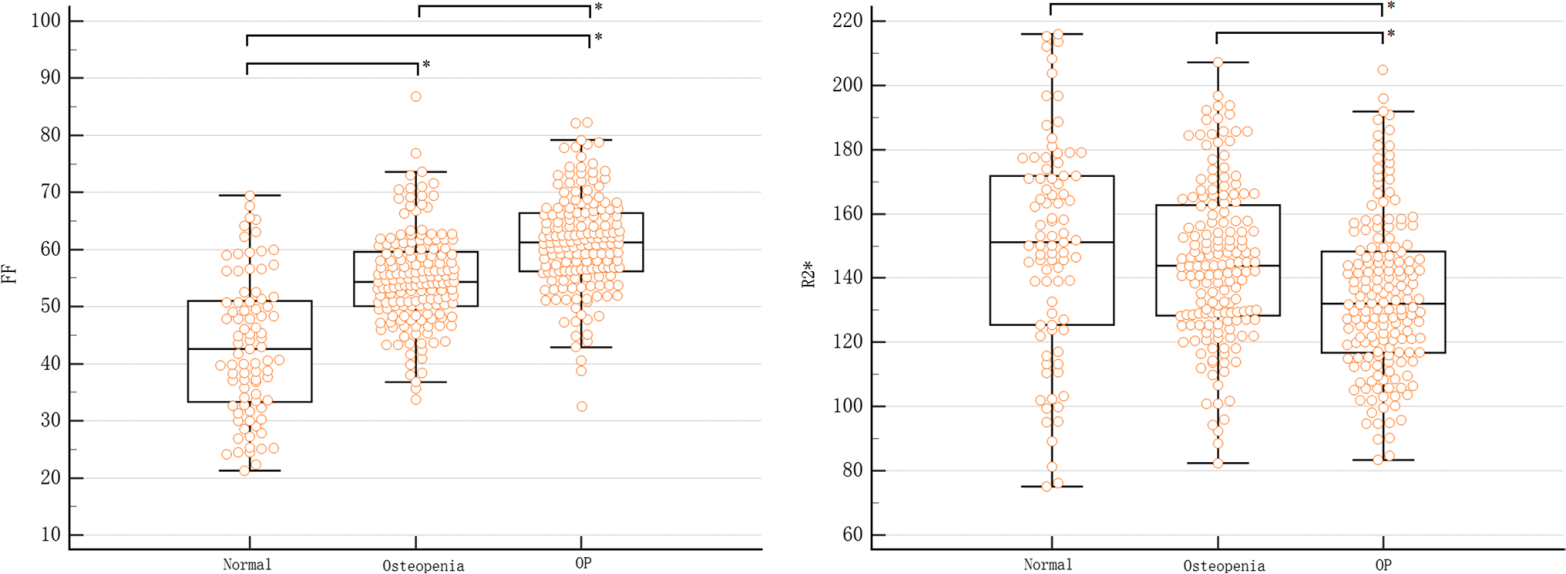
Comparative plots of FF and R2* for groups. *p < 0.05

All 415 vertebrae were divided into three groups according to BMD. The BMD, FF, and R2* values for each group are shown in Table 1. One-way ANOVA revealed that the overall differences were statistically significant in FF and R2* among groups (*F* values of 102.521 and 11.323, both *p* <  0.05). The Tukey-Kramer test showed that the pairwise comparison between groups was also statistically significant, respectively, and the multiple comparisons of them are shown in Fig. 4.

**Table 1:** Measurements of vertebrae in different OP groups (*n* = 415)

	Normal (85)	Osteopenia (164)	OP (166)	Total (415)	*F*	*p*
BMD (mg/cm^3^)	141.06 ± 21.62	98.76 ± 11.44	57.68 ± 16.35	90.99 ± 35.16	801.285	< 0.001
FF (%)	43.06 ± 12.18	54.89 ± 8.45	61.13 ± 8.80	54.96 ± 81.55	102.521	< 0.001
R2* (Hz)	149.71 ± 33.87	145.06 ± 24.95	134.34 ± 24.44	141.72 ± 27.48	11.323	< 0.001

*OP* osteoporosis, *BMD* bone mineral density, *FF* fat fraction

### Correlation analyses

**Fig. 5:**
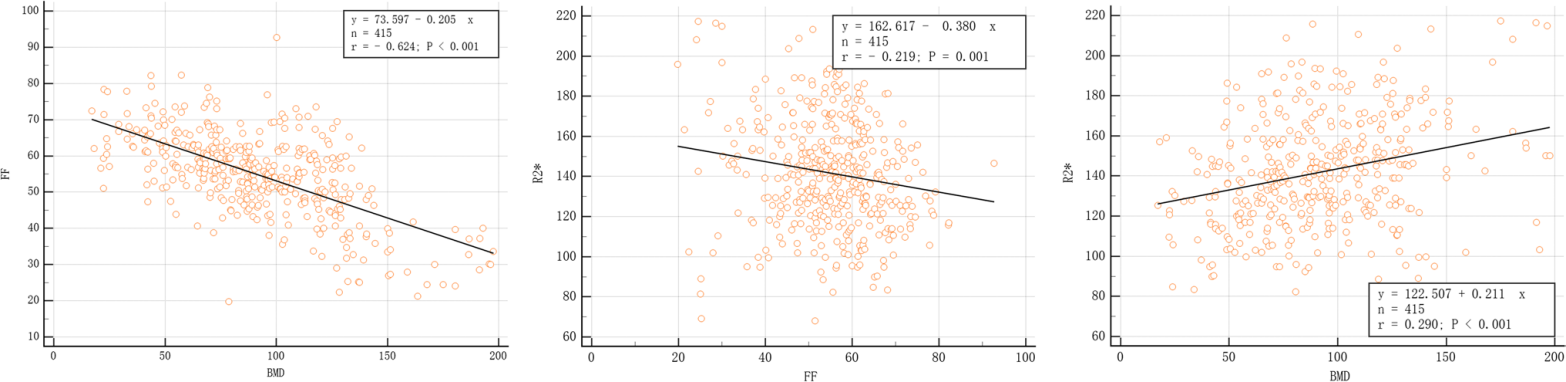
Correlation between R2*, FF, and BMD

Pearson’s test showed a significant correlation between FF and BMD (*r* = −0.624, *p* < 0.001), and there were also weak correlations between R2* and FF and BMD with *r* values of 0.219 and 0.290, respectively, both *p* < 0.001 (Fig. 5).

### Diagnostic test

**Fig. 6:**
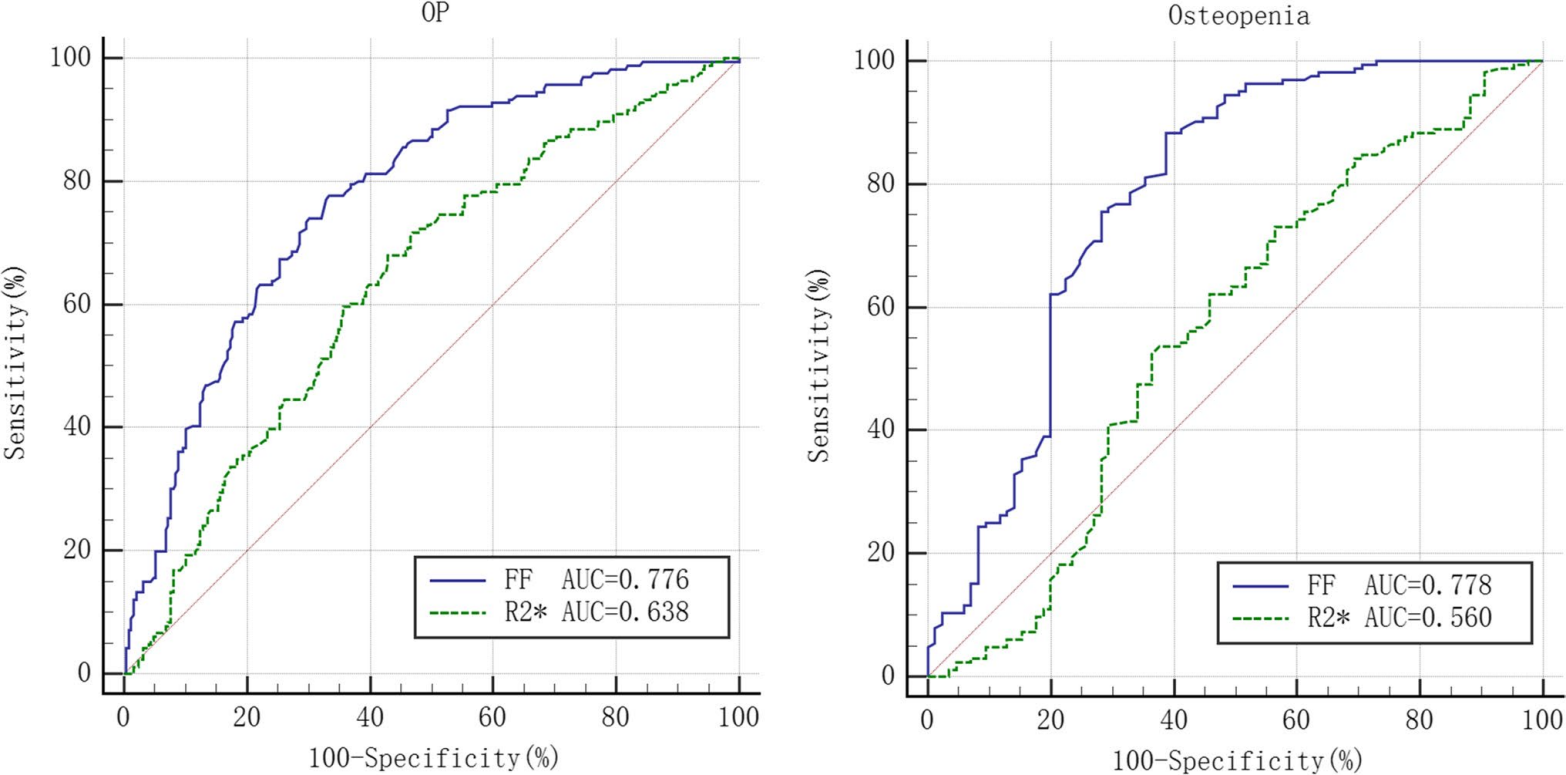
Efficacy for FF and R2* in OP and osteopenia diagnosis

The ROC curves for FF and R2* in the diagnosis of OP and osteopenia are shown in Fig. 6, and the threshold, sensitivity, specificity, AUC, and predictive value are shown in Table 2. The DeLong test showed lower AUCs for R2* than for FF in the diagnosis of OP and osteopenia, with *Z* values of 4.030 and 4.087, respectively, both *p* < 0.001.

**Table 2:** Efficacy for FF and R2* in diagnosis of OP and osteopenia (*n* = 415)

		Threshold	Sensitivity	Specificity	AUC (95%*CI*)	+ PV (%)	- PV (%)
OP	FF (%)	> 55.80	77.71	66.67	0.776 (0.732–0.815)	60.85	81.77
	R2* (Hz)	≤ 142.40	68.07	57.03	0.638 (0.589–0.684)	51.36	72.82
Osteopenia	FF (%)	> 46.30	88.41	61.18	0.778 (0.721–0.828)	60.29	88.79
	R2* (Hz)	≤ 157.70	73.17	53.53	0.560 (0.496–0.623)	51.21	74.95

*FF* fat fraction, *OP* osteoporosis, AUC area under the curve, *+ PV* positive predictive value, *- PV* negative predictive value

## Discussion

In this study, we investigated the relationship between vertebral R2* and FF based on MRI in IDEAL-IQ sequence and BMD based on QCT, and then performed diagnostic experiments. The results revealed a definite but weak linear relationship between R2* with FF and BMD, which has limited value as a diagnostic indicator for OP and osteopenia, but has some potential as a complement to FF and BMD for fine quantification of bone marrow conversion and bone mineral loss.

Bone marrow is a dynamic organ whose composition changes during growth, aging, and multiple disease processes [23]. During childhood, the bone marrow of the vertebral body consists mainly of red marrow, and as the body ages, BMAT accounts for 70% of the bone marrow due to the conversion of most of the red marrow to yellow marrow [24]. It was noted that BMAT has multiple endocrine effects that inhibit osteoblast differentiation and proliferation, leading to decreased bone formation and reduced BMD [23, 25]. This was confirmed in the present study, where FF of the vertebral body showed a significant negative correlation with BMD, and was of value in the diagnosis of OP, similar to what was previously reported [12].

R2* is associated with the deposition of ferritin in the bone marrow, which is located mainly in the red bone marrow [17, 18]. In patients with osteoporosis, the bone mineral content is reduced, the bone trabeculae are thinner, the trabecular space is enlarged, its residual space is filled by a large amount of adipose tissue, and the red bone marrow content is relatively reduced, so R2* correlates with both FF and BMD. In addition, it has been shown that bone is more paramagnetic than bone marrow and that the trabecular-bone marrow interface causes local magnetic field inhomogeneity, which can be measured as T2* (R2*) [19]. In the present study, R2* values of vertebrae were positively correlated with BMD and negatively correlated with FF, which we suggest is related to the widening of the trabecular gap, the reduction of the trabecular-marrow interface, and the fatty conversion of red bone marrow when osteoporosis occurs.

Notably, R2* was weakly correlated with FF and BMD, and FF and BMD were significantly correlated in our study, unlike Watanabe’s study [21], where R2* was correlated with BMD (*r* = 0.602), but FF was not. We believe that the susceptibility of bone mineral loss and bone marrow fat conversion to other factors (e.g., nutritional fluctuations, hormonal changes, and metabolic disorders) [26] contribute to the different findings. The exclusion of metabolic and hematopoietic system diseases other than osteoporosis in our study and the use of point-to-point ROIs in the data measurements reduced the influence of the above-mentioned factors on our findings. In the diagnostic test, the AUC of R2* for both diagnosing OP and osteopenia was not high and lower than that of FF in diagnosing OP, which hardly allowed us to use it as an independent diagnostic indicator for OP. However, it has some advantages in reflecting bone marrow conversion as well as microstructural changes in bone trabeculae, during the development of OP, and can be used as a complement to FF and BMD for fine quantitative assessment of OP.

There were still some deficiencies in this study. First, this study was performed at a single center on a small number of subjects. Studies including larger patient cohorts in multicentric trials will be necessary to further demonstrate the robustness of IDEAL-IQ results. Second, due to software reasons, we did not guarantee that the shape and area of the ROIs were consistent, although they were carefully placed in the first 2/3 of the cancellous bone of the vertebral body, which might also have caused some potential errors.

In conclusion, R2* based on IDEAL-IQ sequences has a definite linear relationship with FF and BMD, but it is not yet sufficient as a separate clinical diagnostic indicator and can be used as a complement to FF and BMD for quantitative assessment of OP.

## Abbreviations

AUC: Area under the curve
BMAT: Bone marrow adipose tissue
BMD: Bone mineral density
FF: Fat fraction
ICC: Intraclass correlation coefficient
IDEAL-IQ: Iterative decomposition of water and fat with echo asymmetry and least-squares estimation
MRI: Magnetic resonance imaging
OP: Osteoporosis
QCT: Quantitative computed tomography
ROC: Receiver operating characteristic
ROI: Region of interest

## Declarations

### Guarantor

The scientific guarantor of this publication is Prof. Yonghong Jiang.

### Statistics and biometry

No complex statistical methods were necessary for this paper.

### Methodology

• prospective

• diagnostic or prognostic study

• performed at one institution
